# Pull-In Effect of Suspended Microchannel Resonator Sensor Subjected to Electrostatic Actuation

**DOI:** 10.3390/s17010114

**Published:** 2017-01-08

**Authors:** Han Yan, Wen-Ming Zhang, Hui-Ming Jiang, Kai-Ming Hu

**Affiliations:** 1 State Key Laboratory of Mechanical System and Vibration, School of Mechanical Engineering, Shanghai Jiao Tong University, Shanghai 200240, China; spondor@sjtu.edu.cn (H.Y.); qmihu@hotmail.com (H.-M.J.); hukaiming@sjtu.edu.cn (K.-M.H.); 2 Department of Mechanical Engineering, University of California Berkeley, Berkeley, CA 94720, USA

**Keywords:** MEMS, suspended microchannel resonators, electrostatic actuation, internal fluid flow, instability, dynamics

## Abstract

In this article, the pull-in instability and dynamic characteristics of electrostatically actuated suspended microchannel resonators are studied. A theoretical model is presented to describe the pull-in effect of suspended microchannel resonators by considering the electrostatic field and the internal fluid. The results indicate that the system is subjected to both the pull-in instability and the flutter. The former is induced by the applied voltage which exceeds the pull-in value while the latter occurs as the velocity of steady flow get closer to the critical velocity. The statically and dynamically stable regions are presented by thoroughly studying the two forms of instability. It is demonstrated that the steady flow can remarkably extend the dynamic stable range of pull-in while the applied voltage slightly decreases the critical velocity. It is also shown that the dc voltage and the steady flow can adjust the resonant frequency while the ac voltage can modulate the vibrational amplitude of the resonator.

## 1. Introduction

Miniaturized beam sensors are significant components of microelectromechanical systems (MEMS) which have extensive applications in information technology, biomedicine, aerospace, etc. [1,2]. In many applications, beam resonators are required to be operated in fluidic environments where the fluid damping degrades the signal-to-noise ratio of measurements and hence limits the application. Burg et al. [3] developed a novel resonator whereby a microchannel was embedded in the microbeam with vacuum outside, called as suspended microchannel resonator. The dissipated energy in the devices was almost identical when liquid or air flowed through the channel and was much lower than that in conventional microcantilevers which were immersed in the same fluid [4]. The good dynamic characteristics ensure a pure resonance which greatly increases the sensitivity of measurements for resonant frequency [4]. As a result, the suspended microchannel resonators have broad applications, including sensing of biomolecules [3,5,6,7], investigation of phase transitions [8], and measurement of the fluid viscosity [9] and density [10,11].

The dynamic characteristics of suspended microchannel resonators are fundamental to the extensive applications [4]. The resonant frequency is a key property and is used to measure the mass of particle flowing in the embedded channel [3]. Burg et al. [3,12] pioneered the suspended microchannel resonators and employed them for particle weighing. They employed the first bending mode of the microbeam in the experiments. Lee et al. [13] pointed out that there existed an intrinsic uncertainty in mass measuring for the first bending mode as the particle approached to the free end of the microbeam. Hence, they used the second bending mode to improve the accuracy. Olcum et al. [14] reported a suspended microchannel resonator that employed multiple bending modes of the microbeam. They found that four bending modes were enough for determining the position and mass of flowing particles with a high speed. By measuring the resonant frequency, the phase transitions of the fluid in the embedded channel can also be studied. Minhyuk et al. [8] firstly investigated the phase transitions using the suspended microchannel resonator. They related the temperature-dependent variations of the frequency to the changes in the density of fluid, from which the phase transition temperatures were determined. In addition to the resonant frequency, the vibration amplitude and the quality factor are also significant dynamic properties and can be employed for measurement. Lee et al. [9] presented two methods for measuring viscosity, one is amplitude-based and the other is quality-factor-based. They found that the amplitude-based scheme was much faster while the quality-factor-based scheme had a better accuracy for viscosity measurement. Khan et al. [10] measured the viscosity of fluid with a high accuracy of 0.025 mPa·s by detecting quality factors of the suspended microchannel resonator. Due to the significance of dynamics of suspended microchannel resonators, some researchers have studied on this topic. Burg et al. [15] discovered the nonmonotonic energy dissipation in suspended microchannel resonators. To interpret the phenomenon, Sader et al. [4,16,17] studied the fluid dynamics of the internal fluid in the embedded channel and presented a theoretical model, which was rigorous and was corroborated by experimental data. The results shown that the quality factor not only non-monotonicaly varied with the increasing of viscosity, but also decreased as the mode number increased. Zhang et al. [18] developed a theoretical model to investigate the dynamic characteristics of suspended microchannel resonators by studying the fluid-structure interactions between the laminar flow and the vibrational microbeam. The instability, frequency variation and energy dissipation were analyzed and discussed.

In the aforementioned references [4,15,16,17,18], the suspended microchannel resonator was regarded as a novel microfluidic device and the effects of intrinsic factors, including fluid viscosity, material of the beam and fluid velocity, on suspended microchannel resonators were studied. However, as one kind of beam resonator, when the suspended microchannel resonator is performed, the actuation mechanism should be taken into account. Beam resonators can be actuated by several ways, including electrostatic actuation [19], piezoelectrical actuation [20], electromagnetical actuation [21] and so on. For suspended microchannel resonators, electrostatic actuation is usually employed [3,22] due to its inherent advantages, including high efficiency, low power consumption, simple structure and quick response [23]. However, electrostatic actuation has an intrinsic instability situation, which is called as the pull-in instability [24,25]. As the applied voltage exceeds a critical value, known as the pull-in voltage, the mechanical restoring force are unable to resist the electrostatic force, hence inducing the collapse of the beam. Pull-in effect is significant for the design and performance of microbeam-based sensors subjected to electrostatic actuation. As a result, many researchers have paid attention to studying the pull-in instability during the past decades. A number of theoretical and numerical methods have been developed for the analysis of pull-in effect, including reduced order models [26,27], finite element method [28], Full-Lagrangian method [29,30], perturbation method [31] and so on.

Electrostatically actuated suspended microchannel resonators are one kind of coupled systems which involve microcantilever, laminar flow, flowing particle and electrical field. Many researchers have paid attention to dynamics of microbeams conveying fluids in the past years [32,33,34,35,36,37,38,39]. Rinaldi et al. [32] initiated the theoretical analysis of miniaturized beam resonators conveying internal fluid flow. They studied the influences of flow velocity on instability, frequency variation and damping using the classical equations for fluid-conveying microbeams presented by Paidoussis [40]. Abbasnejad et al. [36] presented the instability analysis of a fluid-conveying microbeam axially loaded with a pair of piezoelectric layers. They found that imposing voltage difference to piezoelectric layers can increase the critical flow velocity and hence improve the stability. By considering the electrical field, Dai et al. [39] developed a theoretical model to predict the dynamics and pull-in behavior of fluid-conveying microbeams subjected to electrostatic actuation. The results shown that the internal fluid not only influenced the static deflection of the microbeam, but also affected the pull-in voltage. Yan et al. [41] also studied this topic and presented some results. However, differing from the widely studied microbeams which generally contain straight channels, suspended microchannel resonators usually have U-shape channels. Hence, the model characterizing the dynamics of microbeams which contain straight channels is unsuitable for suspended microchannel resonators.

In the paper, a theoretical model for predicting the pull-in effects and dynamic behaviors of suspended microchannel resonators subjected to electrostatic actuation is established by considering the internal fluid and the nonlinear electrostatic force. The pull-in instability, frequency variation and dynamic behaviors are studied and analyzed.

## 2. Model Development

**Figure 1:**
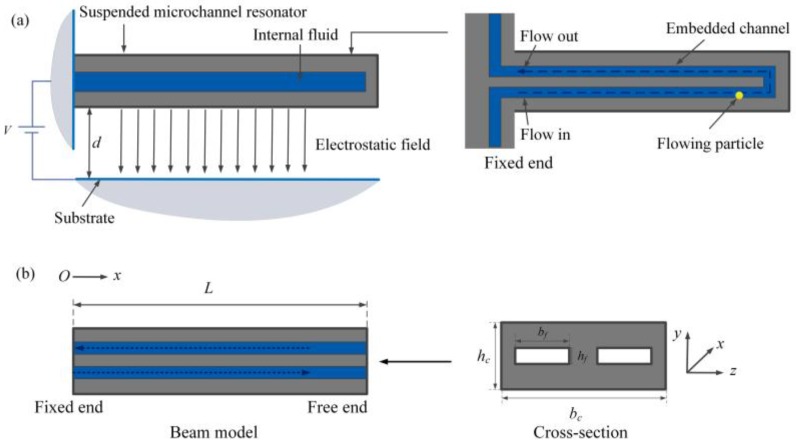
(**a**) Schematic of a suspended microchannel resonator subjected to electrostatic actuation; (**b**) Dimensions of the beam model which is equivalent to the resonator.

As shown in Figure 1a, the suspended microchannel resonator is actuated by the static electricity. Considering the mass of the cross fluid at the end of the channel is much less than the total fluid, the cross flow can be neglected [4,42]. Hence, the embedded channel is regarded as two parallel channels, as shown in Figure 1b. Under this assumption, an electrostatically actuated fluid-conveying microcantilever of uniform thickness $h_{c}$, width $b_{c}$ and length L with two channels of height $h_{f}$ and width $b_{f}$ is considered.

The vibration of the suspended microchannel resonator subjected to the electrostatic field can be modeled as:

(1) $$EI\frac{\partial^{4}w\left(x,t\right)}{\partial x^{4}}+m_{c}\frac{\partial^{2}w\left(x,t\right)}{\partial t^{2}}=F_{elec}\left(x,t\right)+F_{fluid}\left(x,t\right)$$

where *EI* is the flexural rigidity, *x* is the coordinate along the longitudinal direction, $m_{c}$ is the per unit length mass of the resonator, w is the flexural displacement, t is the time, $F_{elec}\left(x,t\right)$ is the electrostatic force and $F_{fluid}\left(x,t\right)$ is induced by the internal fluid flows. The electrostatic force can be given as:

(2) $$F_{elec}=\frac{\varepsilon_{0}b_{c}V^{2}}{2{\left(d-w\right)}^{2}}$$

where $\varepsilon_{0}$ is the permittivity, *V* is the applied voltage, *d* is the initial gap between the microbeam and the substrate. The fluid-induced force $F_{fluid}\left(x,t\right)$ depends on the fluid-structure interactions and was presented by Zhang et al. [18]:

(3) $$F_{fluid}=-2M\left(\frac{\partial^{2}w}{\partial t^{2}}+\frac{6}{5}U^{2}\frac{\partial^{2}w}{\partial x^{2}}\right)$$

where *M* is the fluid mass per unit length. By considering the particle flowing in the channel, the control equation of the system can be expressed as:

(4) $$\begin{array}{l} EI\frac{\partial^{4}w}{\partial x^{4}}+\left(m_{c}+2M+m_{p}\delta \left(x-x_{0}\right)\right)\frac{\partial^{2}w}{\partial t^{2}}+\frac{6}{5}\cdot 2MU^{2}\frac{\partial^{2}w}{\partial x^{2}}+m_{p}\delta \left(x-x_{0}\right)U^{2}\frac{\partial^{2}w}{\partial x^{2}}+\\ 2m_{p}\delta \left(x-x_{0}\right)U\frac{\partial^{2}w}{\partial x\partial t}=\frac{\varepsilon_{0}b_{c}V^{2}}{2{\left(d-w\right)}^{2}} \end{array}$$

where $m_{p}$ is the net added mass of the particle to the fluid, $x_{0}$ is the position and δ is the Dirac function. The previous model given in reference [18] focused on the free vibration of suspended microchannel resonators. The present model extends the previous work by taking the actuation mechanism into account and hence this model can be used to characterize the pull-in phenomenon of suspended microchannel resonators. It is noted that only one particle is taken into account in this model. In practical applications, several particles can be expected to be detected. By neglecting the interactions between the particles, the governing equations for multiple particles can be directly obtained by replacing the term $m_{p}\delta \left(x-x_{0}\right)$ with $\sum_{i=1}^{N_{P}}m_{p}^{i}\delta \left(x-x_{0}^{i}\right)$, where *N_P_* is the number of particles. Without loss of generality, the dynamics when one particle is moving is studied in this paper. The boundary conditions of the cantilever are subjected to:

(5) $$\begin{array}{l} w\left(0,t\right)=0,\;\frac{\partial w\left(0,t\right)}{\partial x}=0\\ EI\frac{\partial^{2}w\left(L,t\right)}{\partial x^{2}}=0,\;EI\frac{\partial^{3}w\left(L,t\right)}{\partial x^{3}}=0 \end{array}$$

For convenience, the non-dimensional form can be expressed as:

(6) $$\eta^{⁗}+\ddot{\eta }+\frac{6}{5}{\hat{U}}^{2}\eta^{\prime \prime }+\delta m_{1}\delta \left(\xi -\xi_{0}\right)\ddot{\eta }+\delta m_{2}\delta \left(\xi -\xi_{0}\right){\hat{U}}^{2}\eta^{\prime \prime }+2\delta m_{3}\delta \left(\xi -\xi_{0}\right)\hat{U}{\dot{\eta }}^{\prime }=\frac{\beta^{2}}{{\left(1-\eta \right)}^{2}}$$

where

(7) $$\begin{array}{l} \eta =\frac{w}{d},\;\xi =\frac{x}{L},\;\tau =\sqrt{\frac{EI}{m+2M}}\frac{t}{L^{2}},\;\hat{U}=\sqrt{\frac{2M}{EI}}UL,\;\delta m_{1}=\frac{m_{p}}{\left(m_{c}+2M\right)L}\\ \delta m_{2}=\frac{m_{p}}{2ML},\;\delta m_{3}=\frac{m_{p}}{\sqrt{2M\left(m_{c}+2M\right)}L},\;\beta^{2}=\frac{\varepsilon_{0}b_{c}L^{4}}{2d^{3}EI}V^{2} \end{array}$$

the spatial and temporal derivatives are given by $\eta^{\prime }=\left(\partial \eta /\partial \xi \right)$ and $\dot{\eta }=\left(\partial \eta /\partial \tau \right)$. The boundary conditions in non-dimensional form can be expressed as:

(8) $$\eta \left(0,t\right)=0,\;\eta^{\prime }\left(0,t\right)=0,\;\eta^{\prime \prime }\left(1,t\right)=0,\;\eta^{\prime \prime \prime }\left(1,t\right)=0$$

The governing equation is derived according to the fluid-structure interactions which consider the fluid viscosity and the velocity profile. As a result, this model is valid for laminar flow which is typical for microscale fluid.

The flexural displacement η(ε,τ) includes two components: the static component and the vibrational component:

(9) $$\eta \left(\varepsilon ,\tau \right)=\eta_{s}\left(\varepsilon ,\tau \right)+\eta_{v}\left(\varepsilon ,\tau \right)$$

The electrostatical force can then been expressed in a Taylor series expansion about $\eta_{v}=0$ by retaining the first two terms:

(10) $$\frac{\beta^{2}}{{\left(1-\eta \right)}^{2}}=\frac{\beta^{2}}{{\left[1-\eta_{s}\left(\varepsilon \right)\right]}^{2}}+\frac{2\beta^{2}}{{\left[1-\eta_{s}\left(\varepsilon \right)\right]}^{3}}\eta_{v}\left(\varepsilon ,\tau \right)$$

The governing equation for static equilibrium can be obtained from Equation (6) by neglecting the time-dependent terms:

(11) $$\eta^{⁗}+\frac{6}{5}{\hat{U}}^{2}\eta_{s}^{\prime \prime }+\delta m_{2}\Delta \left(\xi -\xi_{0}\right){\hat{U}}^{2}\eta_{s}^{\prime \prime }=\frac{\beta^{2}}{{\left(1-\eta_{s}\right)}^{2}}$$

Subtracting the static component from Equation (6), the governing equation for the vibration of the suspended microchannel resonator about its static position can be obtained:

(12) $$\begin{array}{l} \eta_{v}^{⁗}\;+\;{\ddot{\eta }}_{v}+\frac{6}{5}{\hat{U}}^{2}\eta_{v}^{\prime \prime }+\delta m_{1}\Delta \left(\xi -\xi_{0}\right){\ddot{\eta }}_{v}+\delta m_{2}\Delta \left(\xi -\xi_{0}\right){\hat{U}}^{2}\eta_{v}^{\prime \prime }\\ +2\delta m_{3}\Delta \left(\xi -\xi_{0}\right)\hat{U}{\dot{\eta }}_{v}^{\prime }-\frac{2\beta^{2}}{{\left[1-\eta_{s}\left(\varepsilon \right)\right]}^{3}}\eta_{v}=0 \end{array}$$

Equation (12) can be solved to analyze the natural frequency of the system [39] while Equation (6) is used to characterize the pull-in effect and the dynamic behaviors. Since the reduced-order model has been shown to be valid and effective for characterizing the pull-in effect of microbeams and the dynamic characteristics of microbeams conveying internal fluid [18,24,43], the method is directly adopted without further introduction.

The governing Equation (6) can be discretized through the Galerkin procedure. The displacement is expressed as:

(13) $$\eta \left(\varepsilon ,\tau \right)=\sum_{i=1}^{N}\phi_{i}\left(\varepsilon \right)u_{i}\left(\tau \right)$$

where $\phi_{i}$ is the mode shape of the microbeam and $u_{i}$ is the general coordinate. Multiply Equation (6) by $\phi_{n}\left(\xi \right){\left(1-\eta \right)}^{2}$, substitute Equation (13) into the resulting equation, integrate the outcome from ξ = 0 to 1, and obtains:

(14) $$\begin{array}{l} \sum_{i=1}^{N}u_{i}\int_{0}^{1}\phi_{n}\phi_{i}^{iv}d\xi -2\sum_{i,j=1}^{N}u_{i}u_{j}\int_{0}^{1}\phi_{n}\phi_{i}\phi_{j}^{iv}d\xi +\sum_{i,j,k=1}^{N}u_{i}u_{j}u_{k}\int_{0}^{1}\phi_{n}\phi_{i}\phi_{j}\phi_{k}^{iv}d\xi \\ +{\ddot{u}}_{n}-2\sum_{i,j=1}^{N}{\ddot{u}}_{i}u_{j}\int_{0}^{1}\phi_{n}\phi_{i}\phi_{j}d\xi +\sum_{i,j,k=1}^{N}{\ddot{u}}_{i}u_{j}u_{k}\int_{0}^{1}\phi_{n}\phi_{i}\phi_{j}\phi_{k}d\xi +\frac{6}{5}{\hat{U}}^{2}\sum_{i=1}^{N}u_{i}\int_{0}^{1}\phi_{n}\phi_{i}^{\prime \prime }\;d\xi \\ -\frac{12}{5}{\hat{U}}^{2}\sum_{i,j=1}^{N}u_{i}u_{j}\int_{0}^{1}\phi_{n}\phi_{i}\phi_{j}^{\prime \prime }d\xi +\frac{6}{5}{\hat{U}}^{2}\sum_{i,j,k=1}^{N}u_{i}u_{j}u_{k}\int_{0}^{1}\phi_{n}\phi_{i}\phi_{j}\phi_{k}^{\prime \prime }d\xi +\delta m_{1}\phi_{n}\left(\xi_{0}\right)\sum_{i=1}^{N}{\ddot{u}}_{i}\phi_{i}\left(\xi_{0}\right)\\ -2\delta m_{1}\phi_{n}\left(\xi_{0}\right)\sum_{i,j=1}^{N}{\ddot{u}}_{i}u_{j}\phi_{i}\left(\xi_{0}\right)\phi_{j}\left(\xi_{0}\right)+\delta m_{1}\phi_{n}\left(\xi_{0}\right)\sum_{i,j,k=1}^{N}{\ddot{u}}_{i}u_{j}u_{k}\phi_{i}\left(\xi_{0}\right)\phi_{j}\left(\xi_{0}\right)\phi_{k}\left(\xi_{0}\right)\\ +\delta m_{2}{\hat{U}}^{2}\phi_{n}\left(\xi_{0}\right)\sum_{i=1}^{N}u_{i}\phi_{i}^{\prime \prime }\left(\xi_{0}\right)-2\delta m_{2}{\hat{U}}^{2}\phi_{n}\left(\xi_{0}\right)\sum_{i,j=1}^{N}u_{i}u_{j}\phi_{i}\left(\xi_{0}\right)\phi_{j}^{\prime \prime }\left(\xi_{0}\right)\\ +\delta m_{2}{\hat{U}}^{2}\phi_{n}\left(\xi_{0}\right)\sum_{i,j,k=1}^{N}u_{i}u_{j}u_{k}\phi_{i}\left(\xi_{0}\right)\phi_{j}\left(\xi_{0}\right)\phi_{k}^{\prime \prime }\left(\xi_{0}\right)+2\delta m_{3}\hat{U}\phi_{n}\left(\xi_{0}\right)\sum_{i=1}^{N}{\dot{u}}_{i}\phi_{i}^{\prime }\left(\xi_{0}\right)\\ -4\delta m_{3}\hat{U}\phi_{n}\left(\xi_{0}\right)\sum_{i,j=1}^{N}{\dot{u}}_{i}u_{j}\phi_{i}^{\prime }\left(\xi_{0}\right)\phi_{j}\left(\xi_{0}\right)+2\delta m_{3}\hat{U}\phi_{n}\left(\xi_{0}\right)\sum_{i,j,k=1}^{N}{\dot{u}}_{i}u_{j}u_{k}\phi_{i}^{\prime }\left(\xi_{0}\right)\phi_{j}\left(\xi_{0}\right)\phi_{k}\left(\xi_{0}\right)\\ =\beta^{2}\int_{0}^{1}\phi_{n}d\xi \end{array}$$

Equation (14) represents a system including second-order nonlinear ordinary differential equations in terms of the generate coordinates $u={\left(u_{1},u_{2}\cdots u_{N}\right)}^{T}$. The mode shapes $\phi_{i}$ can be given as:

(15) $$\phi_{i}\left(\varepsilon \right)=\left(\cosh\lambda_{i}\varepsilon -\cos\lambda_{i}\varepsilon \right)-\sigma_{i}\left(\sinh\lambda_{i}\varepsilon -\sin\lambda_{i}\varepsilon \right),\;i=1,2,3\cdots$$

where

(16) $$\cosh\lambda_{i}\cos\lambda_{i}+1=0,\;\sigma_{i}=\frac{\sinh\lambda_{i}-\sin\lambda_{i}}{\cosh\lambda_{i}+\cos\lambda_{i}}$$

According to Equations (15) and (16), the integral constants in Equation (14) can be directly obtained. For example, the values of $\int_{0}^{1}\phi_{n}\phi_{i}^{iv}d\xi$ are listed in Table 1.

By introducing a vector $y={\left(y_{1},y_{2}\cdots y_{N},y_{1+N},y_{2+N}\cdots y_{2N}\right)}^{T}$, where $y_{i}=u_{i},\;y_{N+i}={\dot{u}}_{i},\;1\leq i\leq N$, Equation (14) can be transformed to a system composed of 2 N first-order ordinary differential equations. Using the Runge-Kutta method, these equations can be solved and the results can be obtained.

## 3. Results and Discussions

### 3.1. Instability Analysis

**Figure 2:**
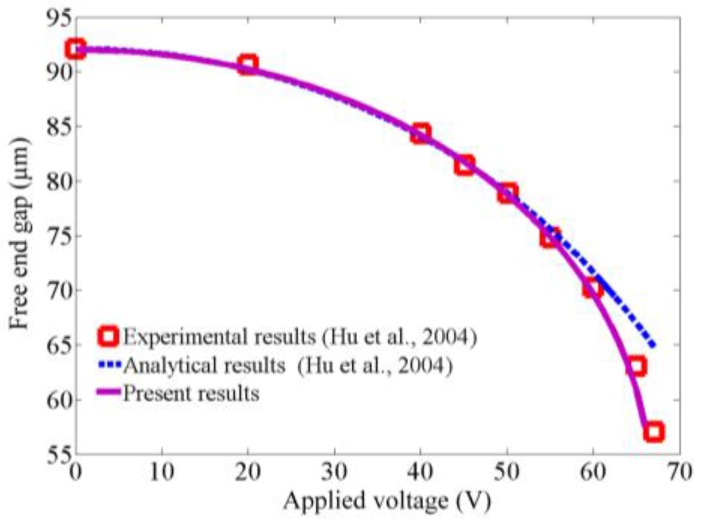
The vatiation of free end gap with the applied voltage.

In this subsection, the pull-in instability and fluid-induced instability are analyzed. To validate the model and the method of solution, consider an electrostatically actuated cantilever with as follows [44]: Young’s modulus is 155.8 GPa, Poisson’s ratio is 0.06, the length of the microcantilever is 20 mm, the width $b_{c}$ is 5 mm, the thickness $h_{c}$ is 57 µm, the initial gap *d* is 92 µm, and the permittivity of air is 8.85 pF/m. The static deflection can be obtained by solving Equation (14) with setting all of the time derivatives equal to zero. Figure 2 shows the results of the end gap versus voltage which are compared with the experimental and analytical results [44]. It can be found that the present results agree well with the reported data, which can verify this model.

**Figure 3:**
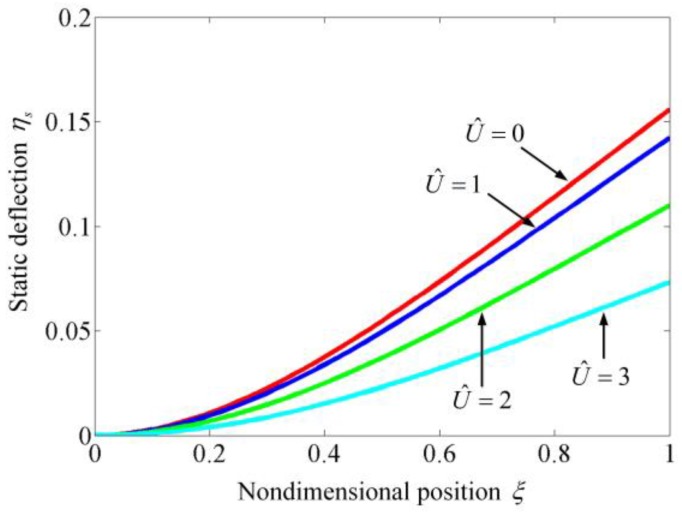
Static deflection of the suspended microchannel resonator for $\beta^{2}=\;1$ and various $\hat{U}$.

**Figure 4:**
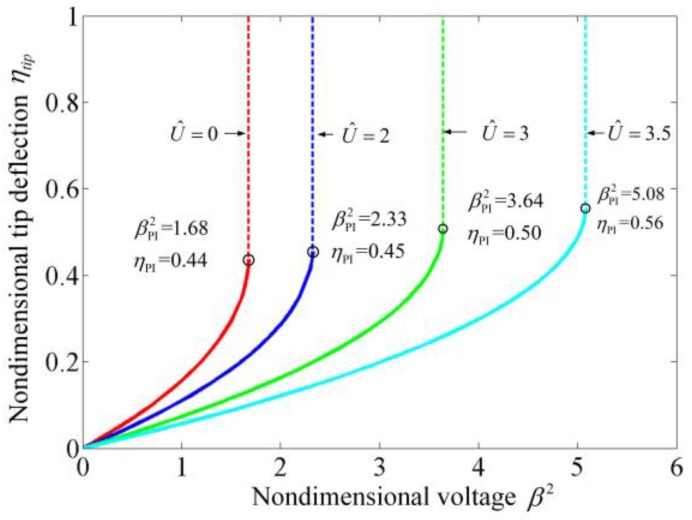
The variation of nondimensional tip deflection with nondimensional voltage for suspended microchannel resonators at different $\hat{U}$.

Figure 3 illustrates the static deflections of the suspended microchannel resonators. It is obvious that as the flow velocity increases, the static deflection decreases. When the flow velocity is zero, because the internal fluid has no stiffness, the fluid has no effect on the static deflection and only the elastic restoring force inhibits the microbeam from bending. As the internal fluid flows with a certain velocity, it induces a centripetal force whose direction is opposite to the electrostatic force. Both the elastic force and the centripetal force keep the microbeam from bending. As a result, as the flow velocity increases, not only the static deflection decreases as shown in Figure 3, but also both the nondimensional static pull-in voltage $\beta_{\mathrm{PI}}^{2}$ and the nondimensional pull-in displacement $\eta_{\mathrm{PI}}$ increase, as illustrated in Figure 4. Figure 4 also demonstrates the static pull-in phenomenon of suspended microchannel resonators and it can be found that the internal fluid can extend the pull-in displacement. It should be noted that the static behaviors of the suspended microchannel resonators are very similar to the ones of electrostatically actuated microbeams conveying fluid which were studied by Dai et al. [39] and Yan et al. [41]. This is because that the static behaviors are studied by solving the governing equations with setting all of the time derivatives equal to zero, which makes the forms of the two governing equations describing the different systems become identical. Table 2 shows the variation of $\beta_{\mathrm{PI}}^{2}$ and $\eta_{\mathrm{PI}}$ with the employed modes in the calculation. It can be found that when the used modes are more than three, the obtained results have no obvious differences. Employing five modes in the calculation can accurately predict the dynamic characteristics.

**Figure 5:**
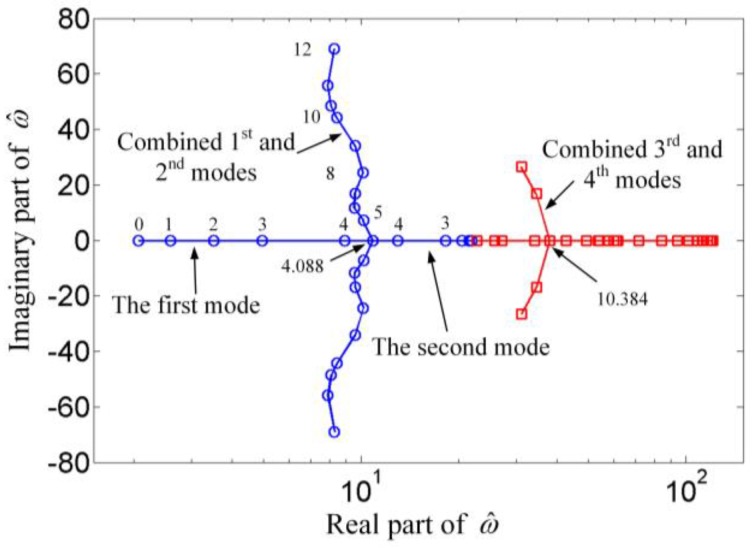
Dimensionless complex frequencies of the suspended microchannel resonator as a function of the dimensionless flow velocity for $\beta^{2}=1.6$.

Although the flow velocity can enhance the static pull-in range, it cannot be increased unlimitedly because the flutter occurs once the velocity exceeds the critical value ${\hat{U}}_{c}$. When $\beta^{2}=0$, the Argand diagram for vibrations of the suspended microchannel resonator has been presented in reference [18]. And the results for $\beta^{2}=1.6$ are illustrated in Figure 5. The parameter $\hat{\omega }$ is the dimensionless complex frequency. The real part of $\hat{\omega }$ is the dimensionless radian frequency, and the imaginary real part represents the damping ratio. It was found that with the increasing of dimensionless velocity $\hat{U}$, the resonant frequency of the first mode increases while the one of the second mode decreases until $\hat{U}$ reaches the critical value ${\hat{U}}_{c}=\;4.088$. It can be found that the Argand diagram for $\beta^{2}\;=\;1.6$ is very similar to the one for $\beta^{2}\;=\;0$. And the critical flow velocity for the first and second mode is almost identical to the value for $\beta^{2}=0$. This phenomenon indicates that the electrostatic force has little effect on the fluid-induced instability of suspended microchannel resonators, at least, for lower values of $\beta^{2}$.

**Figure 6:**
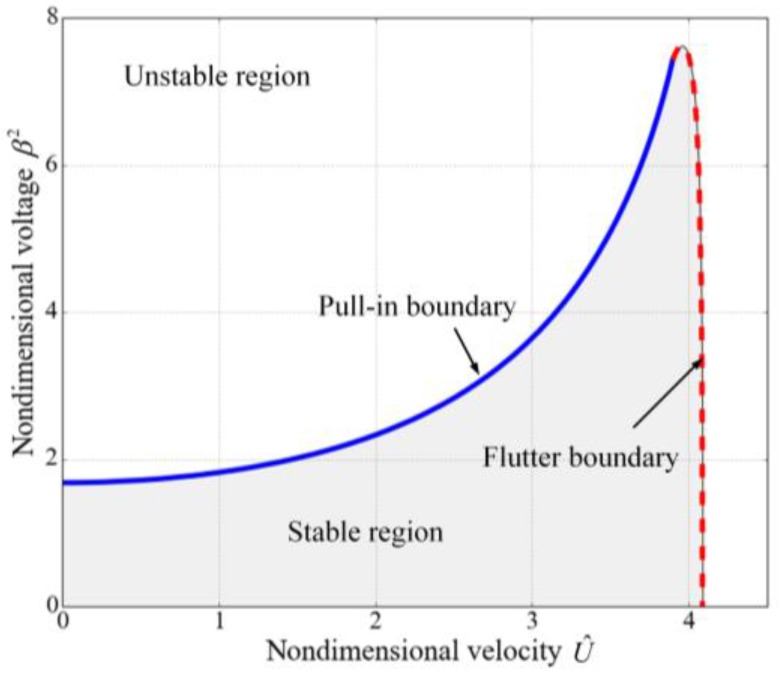
Stability region in the ($\hat{U},\beta^{2}$) plane for the suspended microchannel resonators.

As discussed in the above, the electrostatically actuated suspended microchannel resonators are subjected to both the pull-in instability induced by the electrostatic force and the fluid-induced instability resulted from the internal flow. The values of the nondimensional voltages and the nondimensional velocities cannot exceed some certain values to ensure the stability of the system, as shown in Figure 6. This figure is separated into two sub regions according to different dynamic characteristics. As mentioned in the foregoing, the flow velocity can enhance the static pull-in range, which is illustrated by the pull-in boundary. The applied voltage also affects the critical velocity but the effect is not obvious. As the nondimensional voltage $\beta^{2}$ increases from zero to about 7.484, the critical velocity decreases from 4.088 to 4. For a certain $\beta^{2}$, if $\beta^{2}$ is small (approximately $\beta^{2}<1.679$), the static pull-in instability does not occur and the microbeam loses stability as $\hat{U}$ crosses the flutter boundary. When $\beta^{2}$ is relatively large (approximately $1.679<\beta^{2}<7.436$), the microbeam is subjected to static pull-in instability for small $\hat{U}$, regains stability at a larger value of $\hat{U}$ by crossing the pull-in boundary and again loses stability via flutter at a much larger $\hat{U}$. For very large values of $\beta^{2}$ ($\beta^{2}>7.618$), the system loses stability absolutely. As discussed in the reference [41], the stable regions for the electrostatically actuated microbeams conveying fluid depend on the mass ratio, which is the ratio of the fluid mass per unit length to the structure mass per unit length and characterizes the effects of Coriolis force. However, for the suspended microchannel resonators, the Coriolis forces in the embedded channels cancel out each other due to the opposite flow directions. Hence, the stable region is not related to the mass ratio.

### 3.2. Frequency Shift

**Figure 7:**
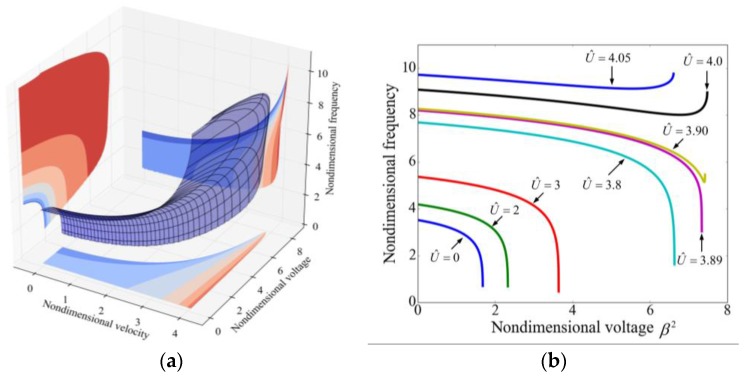

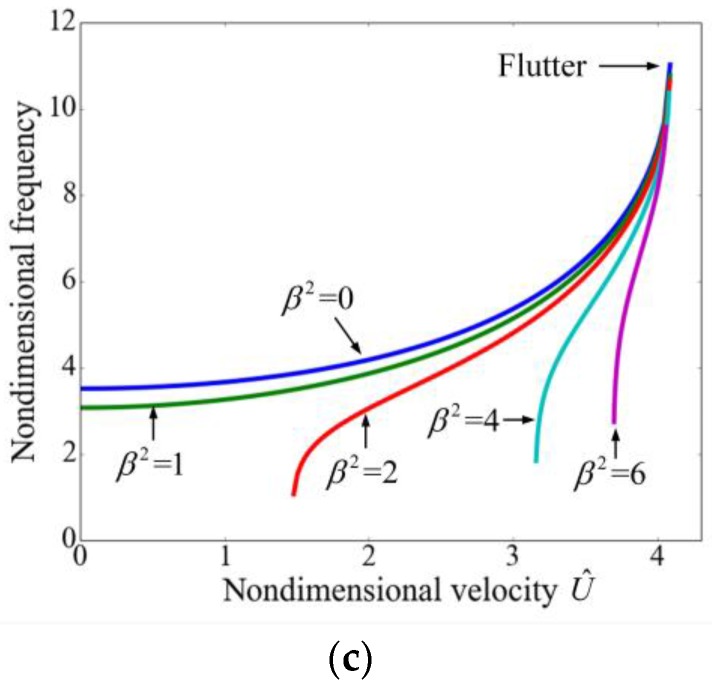
The variation of the fundamental frequency with the nondimensional voltage and the nondimensional velocity. (**a**) The effect of both applied voltage and fluid velocity on the resonant frequency; (**b**) The effect of voltage on the frequency for different fluid velocities; and (**c**) The effect of velocity on frequency for different applied voltages.

The resonant frequency plays a significant role in applications of beam resonators. Figure 7 illustrates the variation of frequency with the applied voltage and the fluid velocity for the fundamental mode. In Figure 7a, the 3D surface intuitively shows the effect of voltage and velocity and it is obvious that both the applied voltage and the internal fluid velocity significantly affect the resonant frequency. The projection of the 3D surface to the bottom depicts the stable boundary, as shown in Figure 6.

Figure 7b demonstrates the variation of frequency with the applied voltage for different velocities. For $\hat{U}<3.9$, the frequency monotonically decreases with the increasing of voltage, and as the voltage approaches to the pull-in voltage, the frequency decreases sharply to zero. This phenomenon has been reported [45] and it is due to the fact that the electrostatic force can be regarded as a spring with negative stiffness. For $\hat{U}\geq 3.9$, as the voltage increases the flutter occurs prior to the pull-in and as a result, the frequency firstly decreases and then increases. The internal fluid flow always makes the frequency increase, as illustrated in Figure 7c. It is noted that for $\beta^{2}\;=\;2$, $\beta^{2}\;=\;4$ and $\beta^{2}\;=\;6$, the values of nondimensional velocity are not started from zero which is due to the instability.

**Figure 8:**
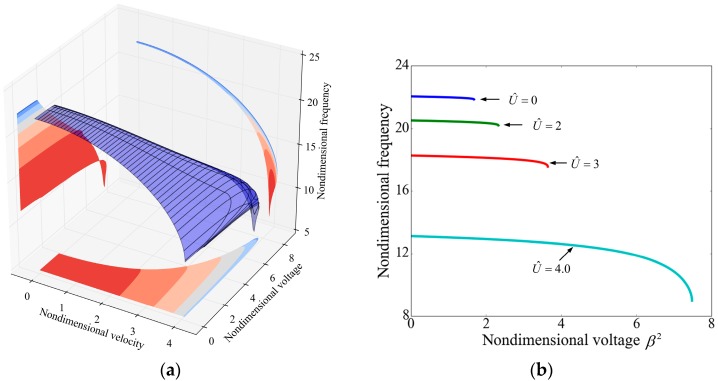

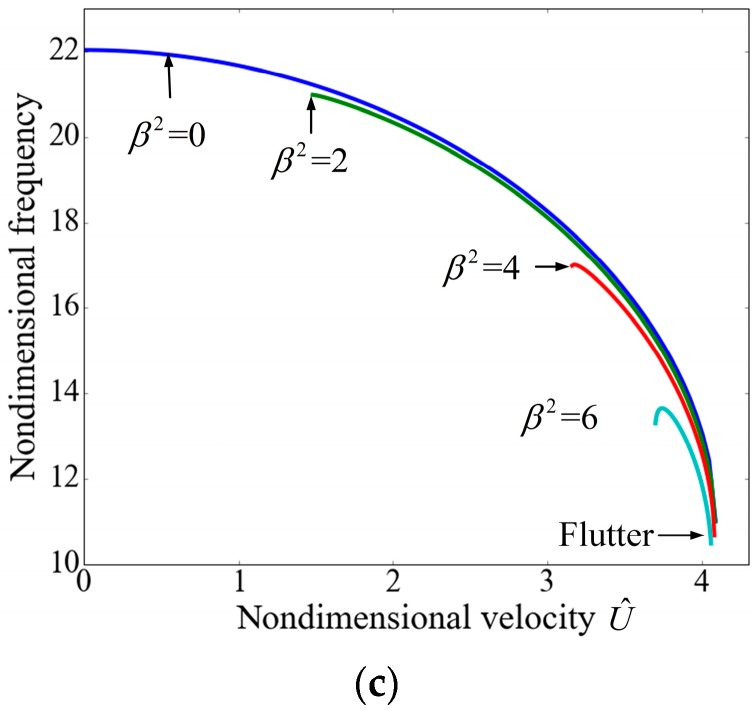
The frequency of the second mode as a function of the nondimensional voltage and the nondimensional velocity (**a**) The effect of both applied voltage and fluid velocity on the resonant frequency; (**b**) The effect of voltage on the frequency for different fluid velocities; and (**c**) The effect of velocity on frequency for different applied voltages.

**Figure 9:**
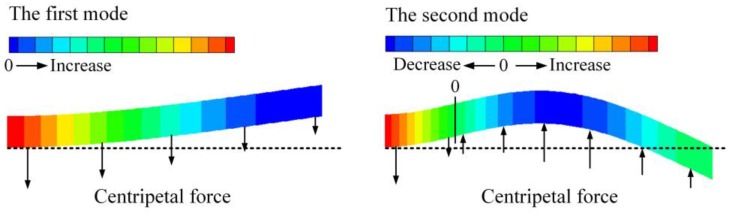
The distribution of the centripetal force for the first and second mode shape.

Figure 8 depicts the frequency shift of the second mode. The applied voltage has no obvious influence on the frequency and the effect of the fluid velocity is contrary to the fundamental mode, which can be attributed to the different mode shapes of the two modes. As shown in Figure 9, for the first mode the centripetal force, induced by the fluid flow and the beam curvature, acts towards to the position of equilibrium and it regards as an additional restoring force. As a result, the effective stiffness of the system is increased by the fluid flow and the resonant frequency increases. For the second mode, the centripetal force acts away from the position of equilibrium as a whole and it works like a “negative” spring. Hence, the frequency of the second mode decreases with the increasing of the flow velocity.

The obtained frequency $\hat{\omega }$ in the above are nondimensional and the dimensional resonant frequency ω can be given by:

(17) $$\omega =\sqrt{\frac{EI}{m+2M}}\frac{1}{L^{2}}\hat{\omega }$$

It is obvious that the frequency increases with both the increasing of stiffness and the decreasing of length or mass. The precision of resonator is improved with the increasing of resonant frequency. By using the parameters of the suspended microchannel resonator developed by Olcum et al. [14], the resonant frequency of the first and second mode can be obtained as 41.32 kHz and 259.0 kHz from the model while the experimental results are 40.48 kHz and 249.1 kHz.

**Figure 10:**
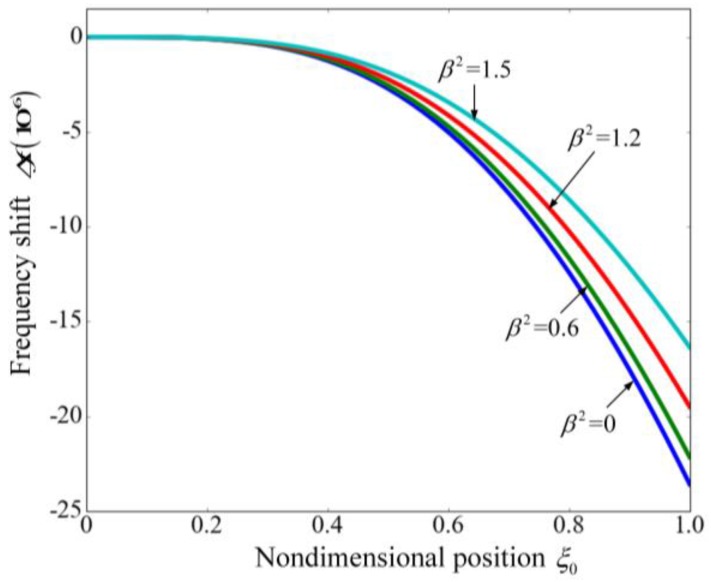
The frequency shift of the fundamental mode as a function of the position of the particle for different nondimensional voltages.

The flowing particle can be regarded as added mass that can induce resonant frequency shift, which underpins the application in measuring the mass of different particles [3,14]. As discussed in the above, the applied voltage plays an important role in the frequency shift. To demonstrate the effect of the voltage and the added mass, Figure 10 shows the frequency shift (Δf) with the position of the particle for different applied voltages. Frequency shift Δf is defined as $f-f_{0}$, where $f_{0}$ is the resonant frequency under the effect of the nondimensional voltage and f is the frequency influenced by both the voltage and the added mass. The parameters are referred to [14]. It can be found that as the applied voltage increases, the variation of the frequency induced by the particle decreases. Because the frequency shift is used to determine the mass of the particle suspended in the fluid [3,14], the decreasing of the frequency shift may reduce the mass resolution of the suspended microchannel resonator. As a result, the applied voltage should not be very large. As listed in Table 3, the normalized frequency shift ($\Delta f/f_{0}$) keeps almost constant as the nondimensional voltage changes. This is because $\Delta f/f_{0}$ is related to the mode shape of the vibrating cantilever [45] and the electrostatic force has no obvious effect on the mode shapes [46]. Consequently, it can be concluded that the normalized frequency shift ($\Delta f/f_{0}$) can accurately characterize the variation of the added mass. For the second mode, both Δf and $\Delta f/f_{0}$ stay almost unchanged as the voltage varies which demonstrates the negligible effect of the electrostatic force.

### 3.3. Dynamic Characteristics

The dynamic pull-in can be defined as the collapse of the microbeam to the substrate under the combined effects of potential and kinetic energies [23]. The kinetic energy can come from the sudden step voltage [47]. In this section, the dynamic pull-in induced by the transient effect of applied voltage is analyzed. The step voltage $\beta^{2}$ is suddenly applied to the resonator at a certain time t = 0. In addition, when the resonator is performed, the applied voltage usually includes two components, the dc component $\beta_{dc}$ and the ac component $\beta_{ac}$. The former is the bias voltage while the latter is the excitation voltage. The dynamic response of the suspended microchannel resonator under the effects of $\beta_{dc}$ and $\beta_{ac}$ is also investigated. To clarify the symbols, β is denoted as the step voltage, $\beta_{dc}$ is denoted as the dc voltage and $\beta_{ac}$ is the ac voltage.

**Figure 11:**
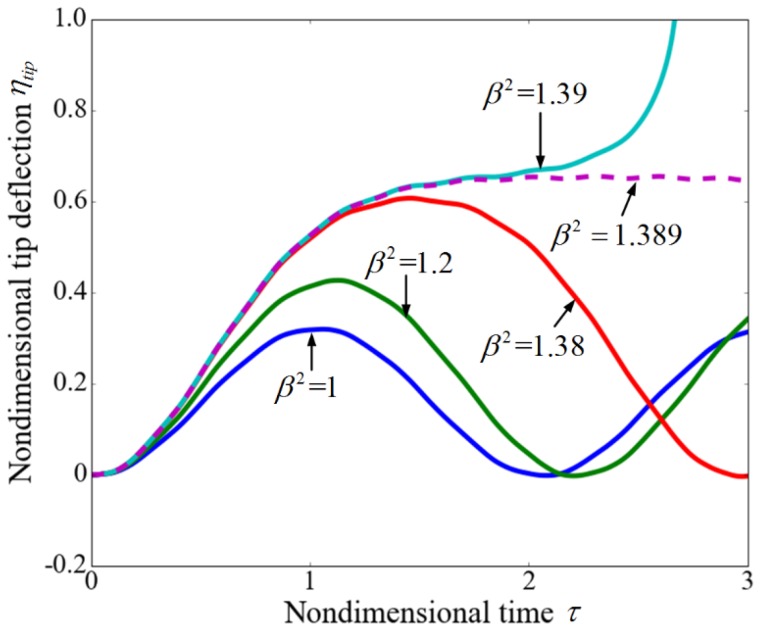
Tip displacement time history of the cantilever for various voltages when the flow velocity equals zero.

Figure 11 shows the transient nondimensional tip deflection $\eta_{tip}$ for different step voltages β when the flow velocity is zero. It is observed that the amplitudes of vibrations increase with the increasing of the applied voltage. When the applied voltage is lower than the dynamic pull-in value $\beta_{\mathrm{DPI}}^{2}$, the microbeam performs a periodic motion. As the voltage exceeds the dynamic pull-in value, the system loses stability and the nondimensional tip deflection reaches unity, which means that the microbeam collapses to the substrate. The results for $\beta_{\mathrm{DPI}}^{2}\;=\;1.389$ and $\beta_{\mathrm{DPI}}^{2}\;=\;1.39$ demonstrate that when the applied voltage is close to the dynamic pull-in value, a small variation in the voltage induces a change in the response. It is noted that the dynamic pull-in voltage ($\beta_{\mathrm{DPI}}^{2}\;=\;1.389$) is smaller than the static pull-in voltage ($\beta_{\mathrm{PI}}^{2}\;=\;1.679$).

**Figure 12:**
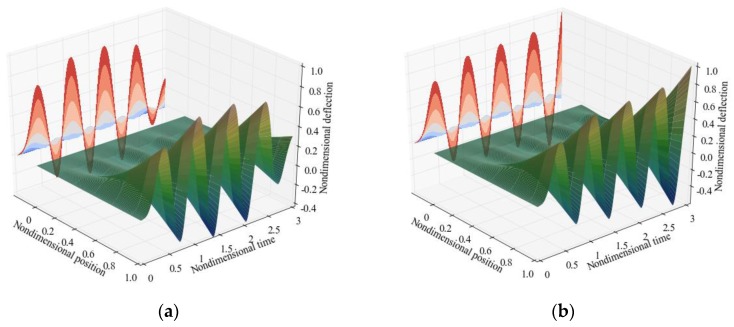
The displacement time history of the whole cantilever for (**a**) $\beta^{2}\;=\;4.243$; and (**b**) $\beta^{2}\;=\;4.244$ when the nondimensional velocity equals 4.

**Figure 13:**
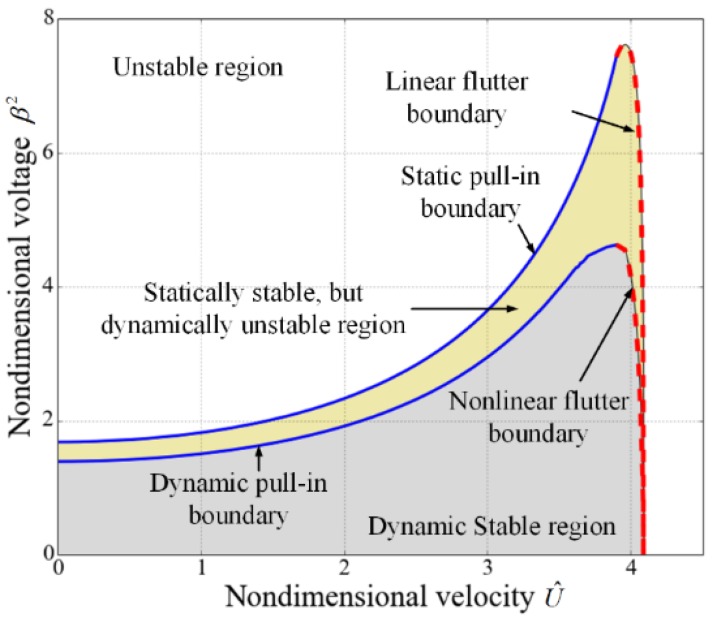
Dynamic and static stability regions in the ($\hat{U},\beta^{2}$) plane for suspended microchannel resonators.

In Section 3.1, the flutter boundary is studied by expanding the nonlinear electrostatic force in a Taylor series expansion and retaining the first order. Now, the nonlinear ordinary differential equations are solved to study the flutter instability. As shown in Figure 12a, when the nondimensional velocity equals 4.0, the system is stable for $\beta^{2}\;=\;4.243$ but becomes unstable for $\beta^{2}\;=\;4.244$. When the flutter occurs, the vibrational amplitude is below unity in the initial several periods but the amplitude constantly increases to unity which means the resonator collapses onto the substrate. It is also noted that the flutter voltage predicted by the dynamic method ($\beta^{2}\;=\;4.243$) is less than the value predicted in Section 3.1 ($\beta^{2}\;=\;7.484$). Figure 13 shows the dynamic and static stability regions and it can be found that the properties of the dynamic stability region are similar to the static one except the area is smaller.

**Figure 14:**
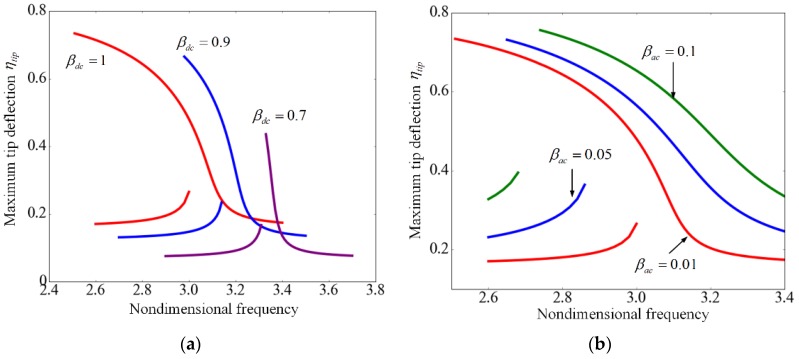
Frequency response curves of suspended microchannel resonators for different (**a**) DC voltages $\beta_{dc}$ with $\beta_{ac}=\;0.01$; and (**b**) ac voltages $\beta_{ac}$ with $\beta_{dc}=\;1.0$ when $\hat{U}=0$.

It can be seen from Figure 14a that for a higher dc voltage the resonant frequency is decreased while the displacement is increased, which has been discussed in the foregoing. Furthermore, as the dc voltage increases, the softening effect becomes stronger. As illustrated in Figure 14b, when the amplitude of the ac voltage $\beta_{ac}$ increases from 0.01 to 0.1, the left and right parts of the frequency response curve moves away from each other. For a certain frequency, the tip deflection increases with the increasing of $\beta_{ac}$, which means that the ac voltage can enhance the vibrational amplitude of the cantilever. In a word, the dc voltage can adjust the frequency while the ac voltage can modulate the amplitude of vibration.

**Figure 15:**
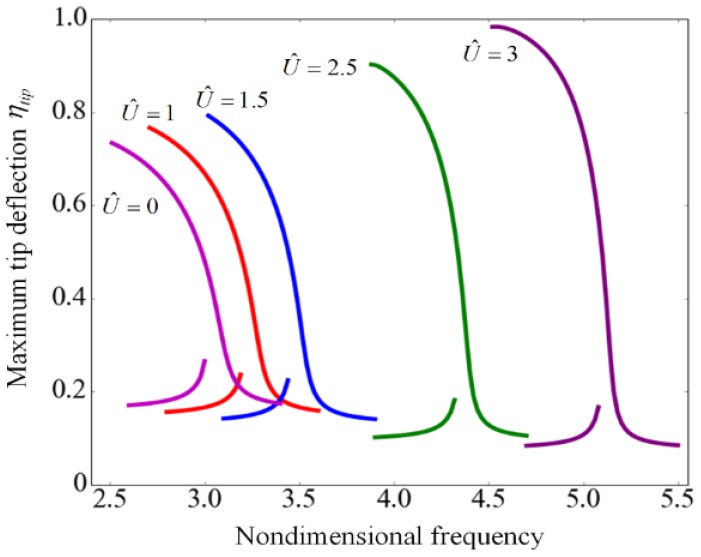
Frequency response curves of suspended microchannel resonators for different nondimensional velocities with $\beta_{dc}=\;1$ and $\beta_{ac}=\;0.01$.

Figure 15 shows the frequency response curves for different flow velocities. As the velocity increases, both the resonant frequency and the maximum deflection of the tip increase. This demonstrates that the flow velocity can not only modulate the frequency but also extend the dynamic stable range of the suspended microchannel resonator, which is attributed to the centripetal force that works as a restoring force as shown in Figure 9.

## 4. Conclusions

A theoretical model is used to describe the pull-in effect and dynamics of the suspended microchannel resonator subjected to electrostatic actuation. The internal fluid and the electrostatic field are considered. The applied voltage affects the frequency shift Δf but has no obvious influence on the normalized frequency shift $\Delta f/f_{0}$, which is beneficial for measurement. The applied voltage can lead to pull-in instability once the voltage exceeds the pull-in value, which can be enhanced by the steady mean flow. The applied voltage is composed of the dc value and the ac value. The former can adjust the frequency while the latter can modulate the vibrational amplitude of the resonator. The steady flow not only leads to frequency shift but also extends the dynamic stable region of pull-in. Furthermore, as the flow velocity reaches the critical one, the flutter occurs and the system loses stability. The results indicate that the suspended microchannel resonator is subjected to both the pull-in instability and the flutter instability. The dynamic and static stable regions are presented by comprehensively considering the two forms of instability.

## Figures and Tables

**Table 1:** The values of the integral constant $\int_{0}^{1}\phi_{n}\phi_{i}^{iv}d\xi$.

	n	1	2	3	4	5
i						
1		$1.236\times 10^{1}$	0	0	0	0
2		0	$4.855\times 10^{2}$	0	0	0
3		0	0	$3.807\times 10^{3}$	0	0
4		0	0	0	$1.462\times 10^{4}$	0
5		0	0	0	0	$3.994\times 10^{4}$

**Table 2:** The nondimensional pull-in voltage and displacement of the suspended microchannel resonator for different modes employed in the calculation at $\hat{U}=3.5$.

The Employed Modes in the Calculation	Nondimensional Pull-In Voltage $\beta_{\mathrm{PI}}^{2}$	Nondimensional Pull-In Displacement $\eta_{\mathrm{PI}}$
One mode	4.66	0.67
Two modes	5.21	0.54
Three modes	5.04	0.56
Four modes	5.11	0.56
Five modes	5.08	0.56

**Table 3:** The frequency shift of the first and second modes when the particle is located at the tip for different nondimensional voltages.

$\beta^{2}$	Δf ($10^{-6}$)		$\Delta f/f_{0}$ (p.p.m)	
	The First Mode	The Second Mode	The First Mode	The Second Mode
0	−23.58	−1.477	−6.705	−6.705
0.6	−22.15	−1.475	−6.708	−6.704
1.2	−19.49	−1.470	−6.720	−6.693
1.5	−16.36	−1.463	−6.742	−6.675
